# Occupational Health and Safety of Finnish Dairy Farmers Using Automatic Milking Systems

**DOI:** 10.3389/fpubh.2016.00147

**Published:** 2016-07-08

**Authors:** Janne P. Karttunen, Risto H. Rautiainen, Christina Lunner-Kolstrup

**Affiliations:** ^1^TTS Work Efficiency Institute, Rajamäki, Finland; ^2^Department of Environmental, Agricultural and Occupational Health, College of Public Health, University of Nebraska Medical Center, Omaha, NE, USA; ^3^The Natural Resources Institute Finland (Luke), Helsinki, Finland; ^4^Department of Work Science, Business Economics and Environmental Psychology, Swedish University of Agricultural Sciences, Alnarp, Sweden

**Keywords:** agriculture, automatic, dairy, farmer, health, milking, occupational, safety

## Abstract

**Introduction:**

Conventional pipeline and parlor milking expose dairy farmers and workers to adverse health outcomes. In recent years, automatic milking systems (AMS) have gained much popularity in Finland, but the changes in working conditions when changing to AMS are not well known. The aim of this study was to investigate the occupational health and safety risks in using AMS, compared to conventional milking systems (CMS).

**Methods:**

An anonymous online survey was sent to each Finnish dairy farm with an AMS in 2014. Only those dairy farmers with prior work experience in CMS were included in the final analysis consisting of frequency distributions and descriptive statistics.

**Results:**

We received 228 usable responses (131 male and 97 female; 25.2% response rate). The majority of the participants found that AMS had brought flexibility to the organization of farm work, and it had increased leisure time, quality of life, productivity of dairy work, and the attractiveness of dairy farming among the younger generation. In addition, AMS reduced the perceived physical strain on the musculoskeletal system as well as the risk of occupational injuries and diseases, compared to CMS. However, working in close proximity to the cattle, particularly training of heifers to use the AMS, was regarded as a high-risk work task. In addition, the daily cleaning of the AMS and manual handling of rejected milk were regarded as physically demanding. The majority of the participants stated that mental stress caused by the monotonous, repetitive, paced, and hurried work had declined after changing to AMS. However, many indicated increased mental stress because of the demanding management of the AMS. Nightly alarms caused by the AMS, lack of adequately skilled hired labor or farm relief workers, and the 24/7 standby for the AMS were issues that also caused mental stress.

**Conclusion:**

Based on this study, AMS may have significant potential in the prevention of adverse health outcomes in milking of dairy cows. In addition, AMS may improve the productivity of dairy work and sustainability of dairy production. However, certain characteristics of the AMS require further attention with regard to occupational health and safety risks.

## Introduction

Occupational injuries and diseases, and other disabling health conditions, are frequent in western agriculture (1, 2). Livestock farmers and workers, particularly those working on dairy farms, are at risk of various adverse health outcomes (3, 4). In addition to acute injuries caused by cattle and the working environment, chronic musculoskeletal conditions result from physical exertion and paced, repetitive, and strenuous working motions and postures in conventional pipeline and parlor milking (4, 5). Respiratory diseases are also frequent among dairy farmers (6).

Investing in and modernizing dairy farm production may have positive effects on the work quality and quantity as well as work safety of dairy farmers (7, 8). Lindahl et al. (4) and Douphrate et al. (5) have reviewed safety practices and interventional efforts to prevent injuries and musculoskeletal disorders in conventional milking systems (CMS).

Studies suggest that automatic (robotic/voluntary) milking systems (AMS) may be of notable help in creating healthier and more attractive working places for future dairy farmers (5, 7). In recent years, AMS have gained much popularity in Finland, other Nordic countries, and elsewhere (9, 10).

The review of Jacobs and Siegford (11) gives a comprehensive description of the technological principles of the AMS. Furthermore, Rodenburg (12) summarizes the current understanding of a robotic barn design, which to some extent, differs from a free-stall (loose housing) barn with a conventional milking parlor.

In the AMS, cows are enticed by concentrate feed to enter the milking stall, where the milking robot cleans the teats, attaches the teat cups, milks the udder on a quarter-basis, detaches the teat cups, and sprays the teats with disinfectant. With regard to work tasks in milking, the role of the dairy worker changes to a great extent from a manual laborer to a system administrator.

The majority of the studies related to AMS focus on the health and welfare of dairy cows, quality and quantity of milk, robotic barn design including cow traffic, and the economy of milk production (11–18). These aspects are important for the improvement of the dairy farmers’ expertise and the profitability of the dairy production. In addition, they may indirectly improve the well-being of dairy farmers and workers as well.

Changing to AMS typically reduces the daily labor requirement in milking and may improve the quality of life through providing more flexibility in work schedules (19–22). According to a survey charting socioeconomic aspects of AMS, it may improve the physical health of dairy farmers, compared to CMS (21). However, there is only limited information on the occupational safety issues regarding the dairy farmers and workers using AMS.

Our survey study had two primary aims. First, we aimed to characterize the key features of the Finnish AMS farms. Second, we aimed to investigate the occupational health and safety risks in using AMS among Finnish dairy farmers compared to their prior experiences in CMS. This information can be used to generate recommendations for the prevention of adverse health outcomes among present and future dairy farmers and workers in Finland and elsewhere.

## Materials and Methods

### Study Setting

Finnish agriculture is based on privately owned family farms. The self-employed farming population includes farmers, spouses, and other salaried family members. They compose over 90% while hired non-family employees compose less than 10% of the permanent workforce on Finnish farms (23). In addition, municipal and private farm relief workers contribute significantly to farm work, especially on dairy farms. In Finland, the statutory farm relief worker services enable farmers with the defined number of livestock (e.g., at least 6 dairy cows, 24 suckler cows, or 90 fattening pigs) to take an annual vacation (26 days in 2016) free of charge while the relief worker takes care of the animal husbandry (24).

In 2014, there were 52,775 farms including 8,370 dairy farms in Finland (25). More than two-thirds (69%) of the dairy farms had a tie-stall (stanchion) barn with a pipeline milking system, and the rest (31%) had a free-stall barn with a milking parlor or an AMS (26). The average herd size was 35 dairy cows; greater in free-stall barns than tie-stall barns, 55 vs. 24 dairy cows, respectively (26).

There were three AMS brands available on the market in Finland at the time of the study. Depending on the AMS brand, one milking robot may operate either one or two milking stalls. According to annually updated sales statistics (9), 904 Finnish dairy farms had AMS with a total of 1,259 milking stalls at the end of 2014. The average number of milking stalls per AMS farm was 1.4. In 2014, the Finnish AMS farms represented about 11% of all dairy farms, but being larger than average, they produced about 25% of the total milk production (9).

### Data Collection

We conducted an online survey of all Finnish dairy farms with an AMS in 2014. Our survey included 22 multiple-choice and open-ended questions charting the key features of the Finnish AMS farms (listed below).

- *Sociodemographic data*: gender and age of the participants (owner-operator).
- *Animal husbandry data*: the number and type of persons contributing to daily animal husbandry, the usage of farm relief workers, workplace orientation, and job guidance of farm relief workers and hired labor, the number of lactating and non-lactating (dry) dairy cows, the presence of rubber flooring in the dairy barn, and prior work experience in CMS (pipeline, parlor, or both).
- *Automatic milking data*: year when AMS was introduced, the number of milking robots, the number of milking stalls, annual milk production, handling method for rejected milk such as colostrum (first milk after calving), type of cow traffic, the number of fetched dairy cows daily, the presence of an operator pit and a closable holding area next to the milking stall(s), training of heifers to use the AMS, incidence of nightly alarms caused by the AMS, and satisfaction with the AMS.

Occupational health and safety risks in AMS vs. CMS were investigated using seven sets of Likert-scale questions with instructions and definitions. The following issues were charted on a five-point scale (*reduces significantly, reduces to some extent, no significant difference, increases to some extent*, and *increases significantly*) augmented with an opt-out choice (*can’t tell*).

- Physical strain in using AMS caused by work that is dynamic (mobile), static, or both – in general and in various body regions.
- Mental stress in general and caused by the specific nature of work in using AMS.
- Risk of occupational injuries caused by various work tasks in using AMS augmented with an open-ended choice.
- Occupational and other work-related diseases caused by different exposures in using AMS.
- Other factors related to AMS.

In addition, the following issues were charted on a three-point scale (*not at all, some*, and *a lot*).

- Physical strain in various work tasks related to AMS augmented with an open-ended choice.
- Mental stress in various issues related to AMS augmented with an open-ended choice.

Our survey was pre-tested by two farmers with an AMS, and some of the questions were edited based on their comments. The Finnish AMS importers forwarded our e-mail cover letter with a link to the survey to their customers, one owner-operator from each AMS farm. One reminder e-mail was sent to all AMS farms.

Our study aimed to compare occupational health and safety risks between AMS and CMS. Hence, only those dairy farmers with at least 1 month of prior work experience in CMS were included in the final analysis. We did not compare specific characteristics (e.g., model, age, or accessories) of the AMS brands or the differences between the brands in our study.

The research team (authors) asserts that this study was performed in accordance with relevant research ethic guidelines based on the Declaration of Helsinki (27). The research team had no access to identifiable information on the study participants. The email invitation to participate stated the purpose of the study and that the online survey was voluntary and anonymous. Informed consent was not used. The companies that emailed the survey invitation to their customers had no access to the responses received by the research team. All responses were stored on a secured server. Ethics approval was not applied as Finnish ethical guidelines do not request it concerning survey studies, which are not interfering with the physical and mental integrity of the study subjects.

### Statistical Methods

The data analysis included examining the means, minimums, and maximums of the continuous variables and categorizing them for further analysis. The frequencies of categorical variables were tabulated, and some variables were reclassified. The Pearson correlation coefficient was calculated for selected variables. The two-tailed chi-square test was used for comparing response proportions of categorical variables including gender, age, the number of persons contributing to daily animal husbandry, the number of automatic milking stalls, and the year of installing the AMS. Only statistically significant differences were reported (*p* < 0.05). The statistical analyses were conducted using SPSS Statistics Version 22 (IBM Corp., Armonk, NY, USA).

## Results

### Characteristics of Farmers and Farms

A total of 228 dairy farmers (131 male and 97 female), one owner-operator per farm, gave usable responses to our survey. The final response rate was 25.2%. Three farmers with no prior work experience in CMS were excluded. Approximately 30% of the responses were obtained after the reminder.

The mean age of the participants was 44 years of age (44 for males and 45 for females). Prior work experience in both conventional pipeline milking and parlor milking was common among the participants (54.8%). Others had work experience in either pipeline milking (35.5%) or parlor milking (9.7%).

The animal husbandry workforce included full-time and part-time owner-operators and hired labor. The majority of the farms (89.9%) had 2–4 persons contributing to daily animal husbandry (range 1–10 per farm), and about half (46.1%) had one or more full-time or part-time hired dairy workers. In addition, 95.2% had a farm relief worker taking care of the dairy cattle during the participants’ annual vacation. Few (1.3%) farms had neither hired labor nor farm relief workers contributing to animal husbandry.

The dairy farms had changed to AMS in 2009 on average (range 2001–2014), and about every tenth farm (12.3%) had installed their AMS in 2014. The responding farms had a total of 316 milking robots operating 321 milking stalls (range 1–5 per farm). The average number of milking stalls per farm was 1.4, and the average number of lactating and non-lactating dairy cows was 82 per farm.

The majority of the farms had one milking stall with 61 dairy cows on average (Table 1). To protect the identity of the two largest farms with four and five milking stalls, their production-specific information is not reported. The number of dairy cows per farm was significantly and positively correlated with both the number of milking stalls (*r* = 0.90, *p* < 0.001) and with the annual milk production per farm (*r* = 0.94, *p* < 0.001).

**Table 1:** **Number of AMS farms, milking stalls, dairy cows, and annual milk production per farm in 2014**.

AMS farms (Frequency)	Milking stalls per farm (Frequency)	Dairy cows^a^ per farm (Frequency)		Annual milk production per farm (Million liters)	
		Average	Range	Average	Range
155	1	61	25–85	0.568	0.150–0.838
56	2	110	62–150	1.021	0.480–1.546
15	3	160	115–200	1.444	1.000–2.010
2	4–5	–	–	–	–

*AMS, automatic milking system*.

*^a^Includes both lactating and non-lactating (dry) dairy cows*.

### Occupational Health and Safety Risks in AMS

#### Physical Strain

**Figure 1:**
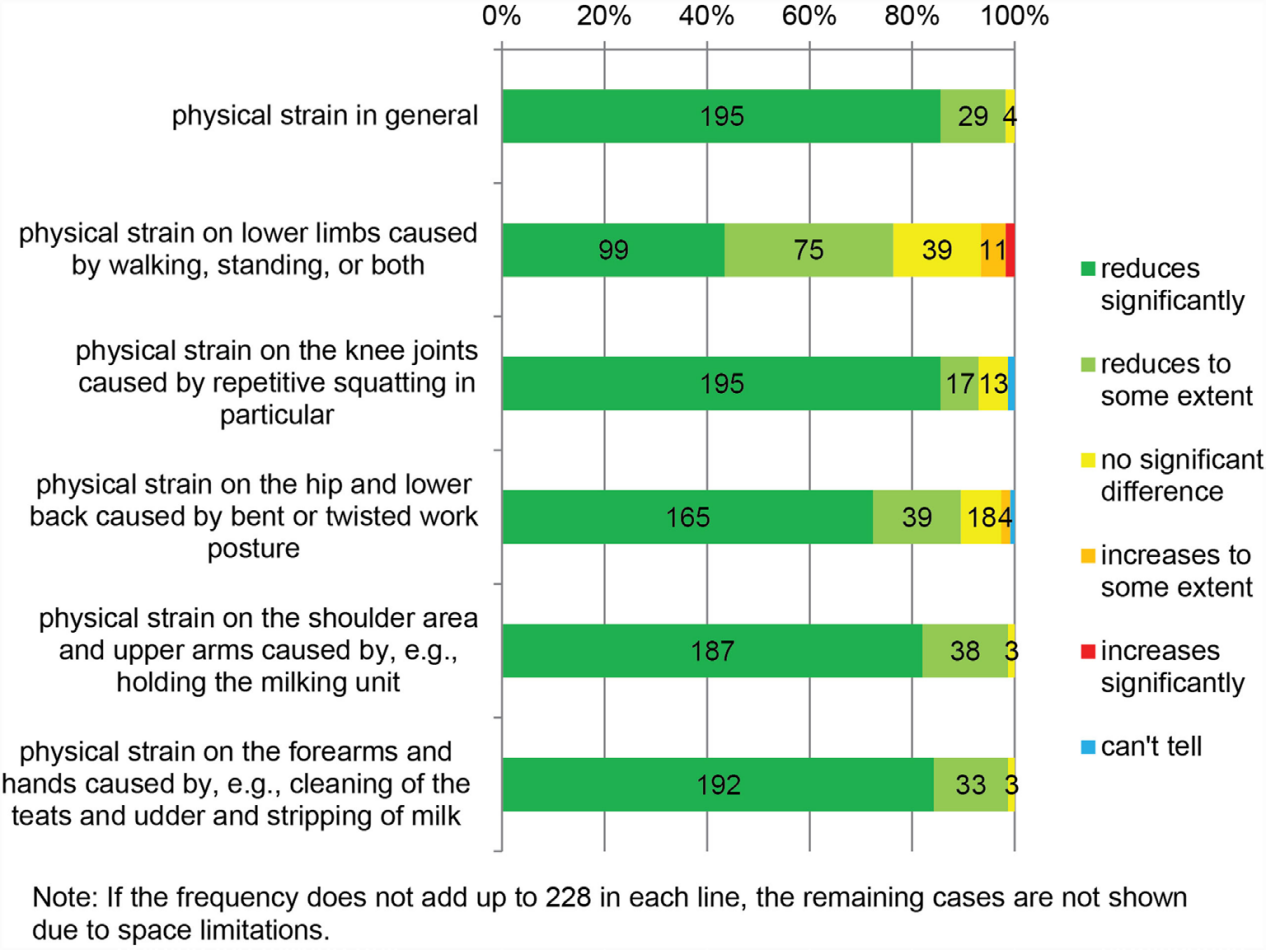
**Physical strain in automatic milking compared to conventional milking (*N* = 228)**.

The dairy farmers’ opinions regarding the perceived physical strain in using AMS, compared to CMS, are shown in Figure 1. Nearly all participants (98.2%) found that AMS reduced the physical strain in general. Few found no significant difference, and none found increased physical strain after changing to AMS.

Furthermore, our survey included five questions regarding the perceived physical strain in various body regions. The majority of the participants found reduced physical strain in all body regions after changing to AMS. The reduction was most evident on the knee joints, forearms, and hands as well as the shoulder area and upper arms. Some farmers found no significant difference, and few found increased physical strain especially in lower limbs or in the hip and lower back when using AMS, compared to CMS.

Compared to females, greater proportion of male farmers reported reduction of physical strain on the lower limbs from walking, standing, or both when using AMS (85.5 vs. 63.9%) (chi-square test, *p* = 0.018).

Less than half of the participants (42.5%) had rubber covering on one or more of the following areas inside the barn: feed alleys next to the feed table(s), manure alleys between the free stalls, and holding area next to the automatic milking stall(s). The presence (or absence) of rubber covering was not associated with either physical strain or occupational injury risk in our study.

Dairy farmers were also asked to estimate the physical strain in seven work tasks related to AMS (Table 2). Daily handling of rejected milk caused some or a lot of physical strain among 66.2% of the dairy farmers. The rejected milk from the AMS was led to either plastic buckets (volume 20 l) or stainless steel buckets (volume 25–30 l), carried away, and emptied manually every day on 85.5% of the farms. Some farms (12.3%) had a specific milk line for rejected milk leading to a larger container. Few farms had both buckets and a line for rejected milk.

**Table 2:** **Perceived physical strain in work tasks related to automatic milking (*N* = 228)**.

Work task	Perceived physical strain		
	Not at all	Some	A lot
	Frequency (%)^a^	Frequency (%)	Frequency (%)
Daily handling of rejected milk^b^	77 (33.8)	141 (61.8)	10 (4.4)
Daily cleaning of the AMS	131 (57.5)	96 (42.1)	1 (0.4)
Fetching cows to the milking stall	163 (71.5)	64 (28.1)	1 (0.4)
Work with the computer	172 (75.4)	53 (23.2)	3 (1.3)
Manual attachment of the teat cups	188 (82.5)	37 (16.2)	3 (1.3)
Daily tasks in the milk room	194 (85.1)	34 (14.9)	–
General observation of the AMS	206 (90.4)	22 (9.6)	–

*^a^Percentages may not horizontally add up to 100.0 due to rounding*.

*^b^Colostrum milk or milk that contains antibiotic residues, excess blood, or somatic cells*.

*AMS, automatic milking system*.

In addition, daily cleaning of the AMS caused physical strain to 42.5% of the participants. This work task includes surface cleaning of the milking robot, teat cups, milk hoses, and automatic milking stall several times per day using a water hose and a brush.

Daily cleaning of the AMS may be conducted either by standing on the same level of the floor where the milking stall is located or by using an operator pit. In our study, 40.8% of the farms had an operator pit located next to the milking stall. The average depth of these pits was about 0.50 m (range 0.20–1.20 m), and 69.9% of the pits were partially surrounded by safety railings. However, the presence or absence of the operator pit was not associated either with physical strain or occupational injury risk in our study.

Management of the daily cow traffic related to AMS was another issue causing physical strain to many farmers (28.5%). The majority of the farms (77.6%) had free cow traffic, and 78.0% of them had a closable holding area next to the milking stall. Guided cow traffic, where a selection gate guides dairy cows with milking permission to the enclosed holding area, was found on 22.4% of the farms.

Most farmers (75.5%) with one automatic milking stall had to fetch fewer than five individual dairy cows daily. Many (23.2%) had to fetch 5–10 cows and some (1.3%) more than 10 cows each day. The average number of cows fetched daily was higher on farms with two or three stalls. Male farmers reported more often that fetching cows to the milking stall caused them physical strain (34.4 vs. 20.6%) (chi-square test, *p* = 0.023).

In addition to the work tasks listed in Table 2, training heifers to use the AMS, manual handling of the detergent and disinfectant containers, and repair and maintenance of the AMS were named as tasks causing physical strain.

#### Mental Stress

**Figure 2:**
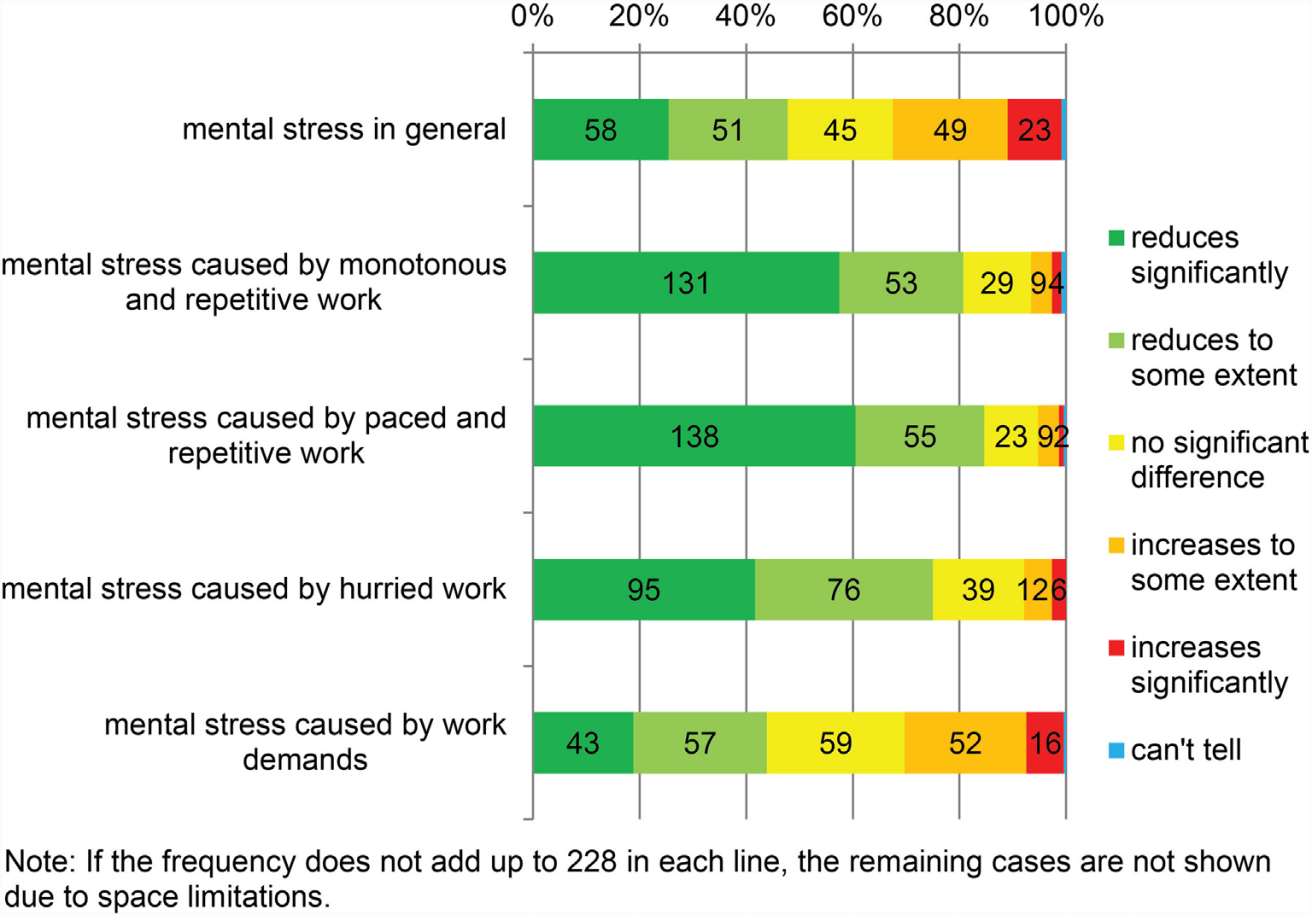
**Mental stress in automatic milking compared to conventional milking (*N* = 228)**.

Participants’ opinions regarding the perceived mental stress after changing to AMS are shown in Figure 2. Approximately half (47.8%) found that AMS reduced their mental stress in general. No significant difference in mental stress was reported by 19.7%, and 31.6% stated that their mental stress had increased.

Four questions addressed perceived mental stress caused by the nature of work using AMS. Mental stress from work demands in AMS (vs. CMS) varied among the participants. However, the majority found that changing to AMS had reduced monotonous, repetitive, paced, and hurried work in milking.

Compared to their peers with longer experience using AMS, those who had installed their AMS in 2014 stated more often that AMS had reduced their mental stress in general (71.4 vs. 45.5%) (chi-square test, *p* = 0.013). Further questions addressed eleven aspects of mental stress (Table 3). The majority (93.4%) mentioned one or more AMS-related issues causing (some or a lot of) mental stress. Three issues in particular emerged in the responses: nightly alarms caused by AMS, trusting farm relief workers and/or hired labor to manage milking with the AMS, and taking care of the 24/7 standby for the AMS.

**Table 3:** **Perceived mental stress in issues related to automatic milking (*N* = 228)**.

Work task	Perceived mental stress		
	Not at all	Some	A lot
	Frequency (%)^a^	Frequency (%)	Frequency (%)
Nightly alarms caused by the AMS	65 (28.5)	117 (51.3)	46 (20.2)
Trusting the farm relief workers, hired labor, or both to manage with the AMS	74 (32.5)	118 (51.8)	36 (15.8)
Taking care of the 24/7 standby for the AMS	110 (48.2)	96 (42.1)	22 (9.6)
Occasionally long work days	131 (57.5)	73 (32.0)	24 (10.5)
Dependency on the timeliness and proficiency of the hired maintenance of the AMS	135 (59.2)	75 (32.9)	18 (7.9)
No clear end for the work day	140 (61.4)	68 (29.8)	20 (8.8)
Trusting the skills of the family members to manage with the AMS	146 (64.0)	75 (32.9)	7 (3.1)
Trusting the operational reliability of the AMS	152 (66.7)	66 (28.9)	10 (4.4)
Alarms caused by the AMS during waking hours	166 (72.8)	60 (26.3)	2 (0.9)
Trusting one’s own skills to manage with the AMS	188 (82.5)	37 (16.2)	3 (1.3)
Work with the computer	199 (87.3)	28 (12.3)	1 (0.4)

*^a^Percentages may not horizontally add up to 100.0 due to rounding*.

*AMS, automatic milking system*.

Nightly AMS alarms caused mental stress to 71.5% of the participants. The majority (87.3%) had none or few nightly alarms per month, and others had nightly alarms at least weekly (11.8%) or almost every day (0.9%).

Trusting the farm relief workers and/or hired labor to manage with the AMS caused mental stress to 67.6% of the farmers. The majority of them (81.1%) stated that these external workers were given workplace orientation and job guidance orally, and that comprehensive written instructions were available. Other farmers (18.9%) had little or no written instructions.

Several farmers (51.7%) experienced mental stress from the 24/7 standby required for managing the AMS. Dependency on the timeliness and proficiency of hired maintenance of the AMS also caused mental stress, which was reported more commonly by female farmers than males (50.5 vs. 33.6%) (chi-square test, *p* = 0.010). In addition to the issues listed in Table 3, power failures and high repair and maintenance costs were mentioned as causing mental stress.

#### Occupational Injury and Disease Risk

**Figure 3:**
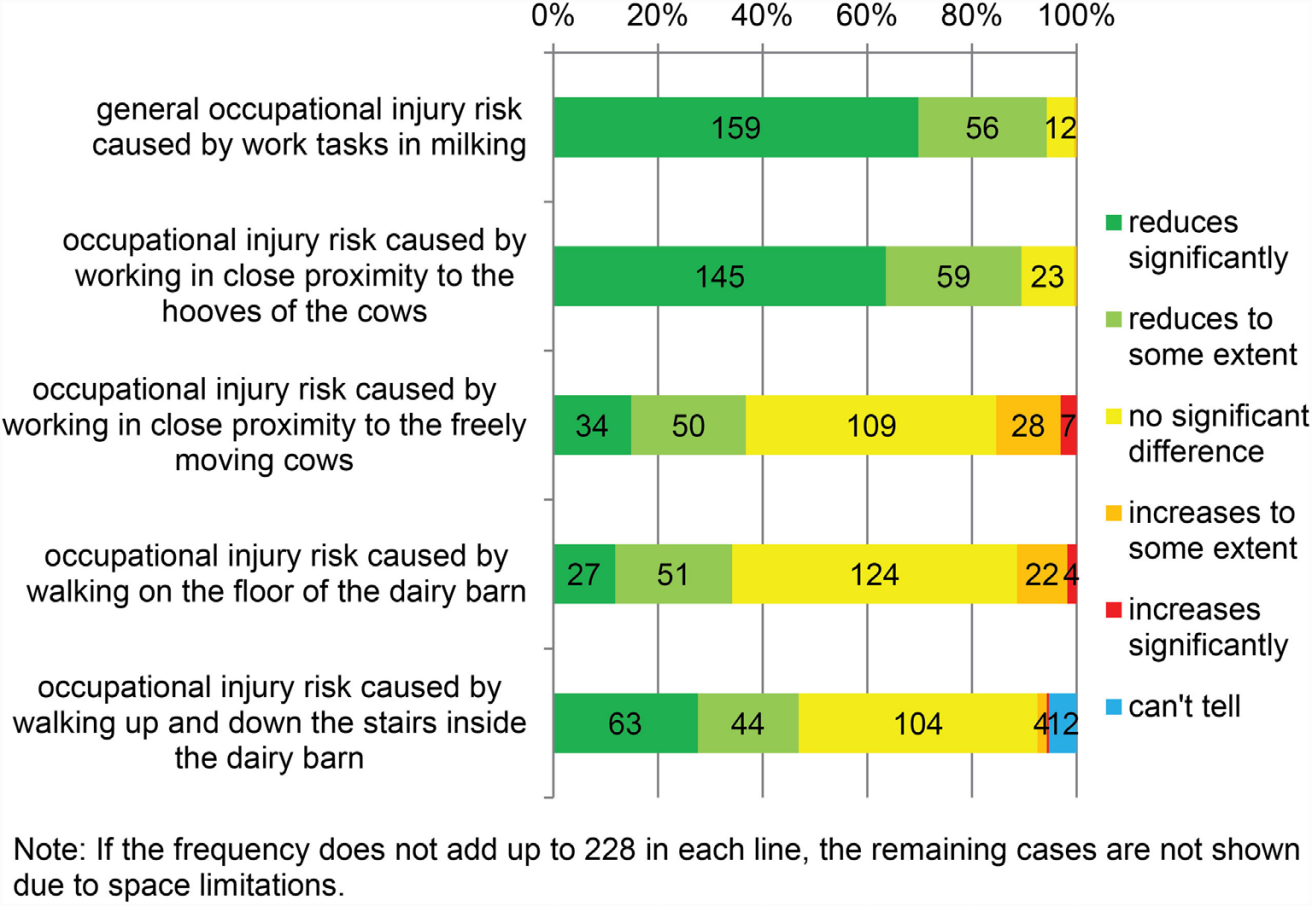
**Occupational injury risk in automatic milking compared to conventional milking (*N* = 228)**.

The dairy farmers’ opinions on the perceived occupational injury risks in using AMS, compared to CMS, are shown in Figure 3. The great majority of them (94.3%) found that AMS reduced injury risk in general. The majority (89.5%) also reported reduced injury risk caused by working in close proximity to the hooves of the dairy cows. However, working in close proximity to the freely moving cows and walking up and down the stairs and on the floor inside the barn divided the participants’ views. These issues may relate to both milking and to animal husbandry in general. The minority of the farmers (34.2–46.9%) reported reduced injury risk, whereas about half of the farmers (45.6–54.4%) saw no difference between AMS and CMS. Only few (11.4–15.4%) perceived that the injury risk had increased after changing to AMS. There was a significant gender difference in perceived reduction in injury risk from working in close proximity to the freely moving cows (males 42.7% vs. females 28.9%; chi-square test, *p* = 0.033).

In addition to the issues listed in Figure 3, the majority of the participants (73.2%) responded to the open-ended question and described an injury risk related to AMS. Most of them (89.8%) mentioned a task where the worker had to work in close proximity to the cattle. The most commonly mentioned work task (89 responses) was training of heifers and cows to use the AMS. Heifers were not trained to use the AMS before their first calving on 49.1% of the farms, while 31.6% reported training all heifers, and the rest trained some of the heifers.

Other commonly mentioned hazardous work tasks were assisting the AMS and medication and grouping of the cattle. Handling the detergent and disinfectant containers were brought up as potential injury risks as well.

**Figure 4:**
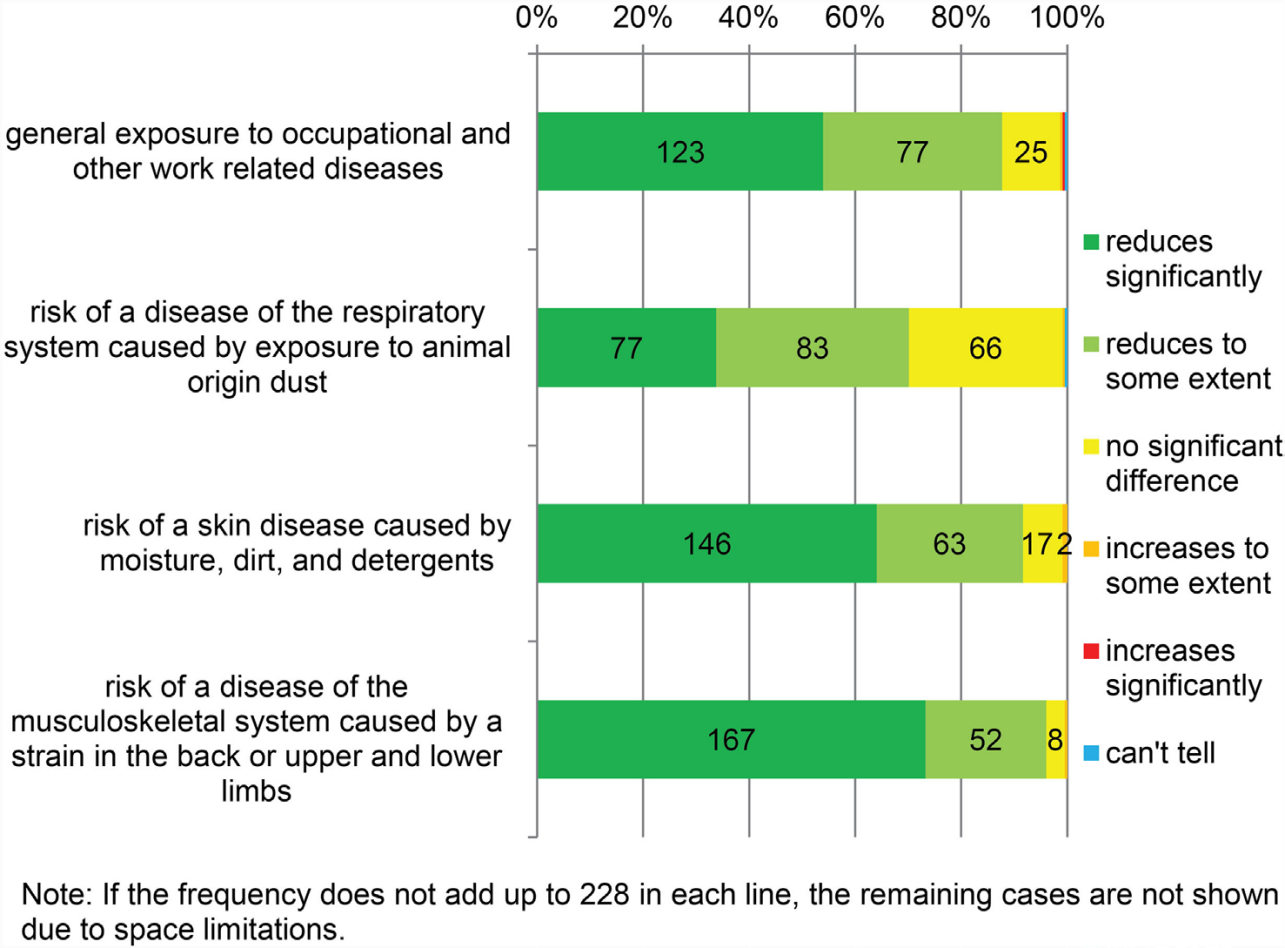
**Occupational and other work-related disease risk in automatic milking compared to conventional milking (*N* = 228)**.

The participants’ opinions regarding the perceived occupational disease risk after changing to AMS are shown in Figure 4. The majority (87.7%) found that AMS reduced the general exposure to occupational and other work-related diseases. Similarly, the majority found that AMS had reduced specific risks of respiratory diseases, skin diseases, and musculoskeletal symptoms compared to CMS (70.2, 91.7, and 96.1%, respectively). Only few (0.9%) perceived that the risk of occupational diseases had increased after changing to AMS. Several farmers (28.9%) saw no difference in the risk of respiratory diseases in using AMS, compared to CMS.

#### Other Factors Related to AMS

**Figure 5:**
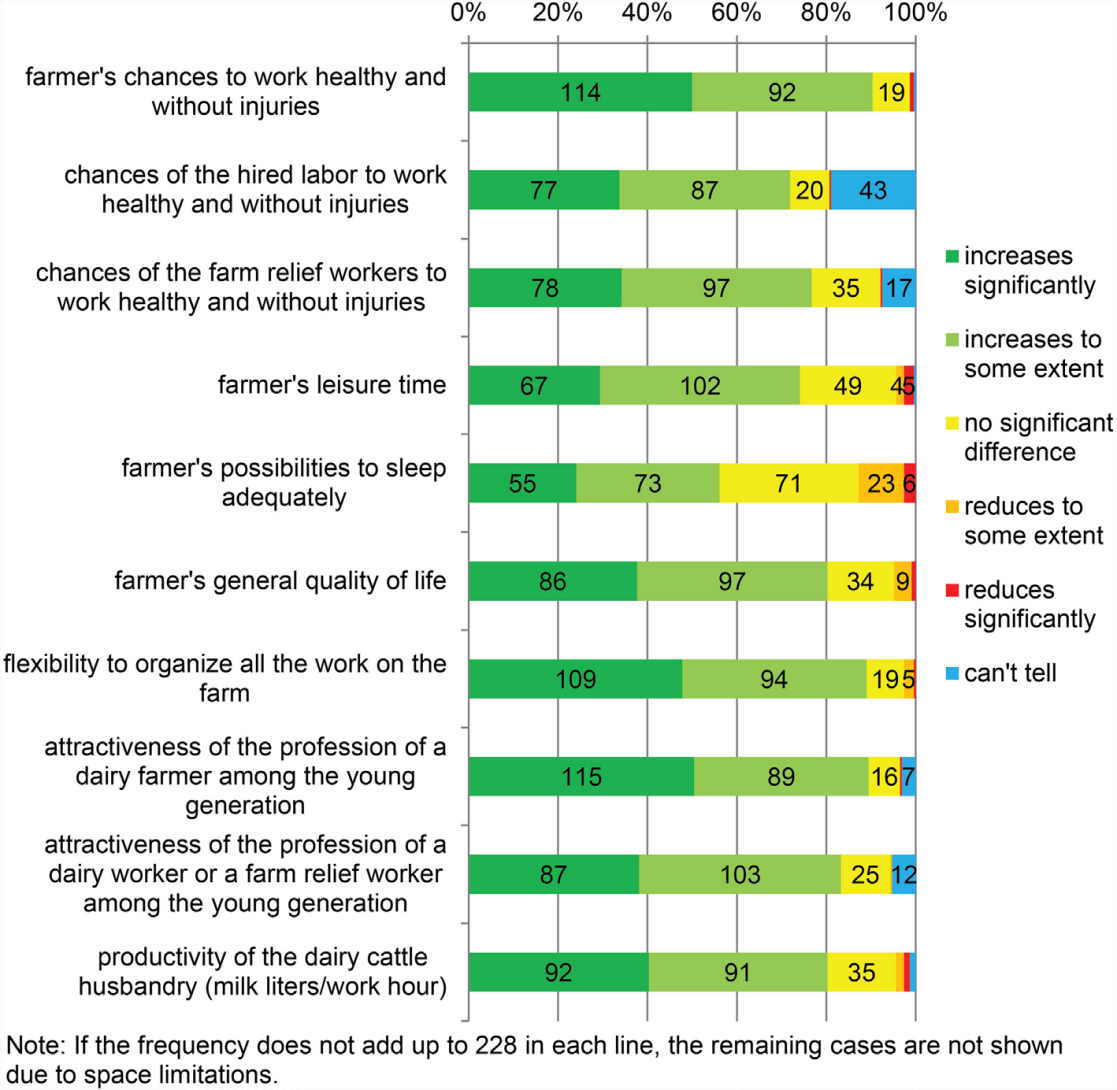
**Other factors in automatic milking compared to conventional milking (*N* = 228)**.

Several other factors related to AMS vs. CMS are shown in Figure 5. The majority of the participants (≥74.1%) found that AMS had brought flexibility to the organization of farm work, and it had increased leisure time, quality of life, productivity of dairy work, and the attractiveness of dairy farming among the younger generation. Furthermore, the majority (≥71.9%) stated that changing to AMS had increased the dairy farmer’s own chances as well as the chances of the hired labor and farm relief workers to work healthy and without injuries. However, the perceived possibilities to get adequate sleep after changing to AMS varied among the participants.

The majority of the participants (93.0%) had no intentions to change their current AMS brand or to change from AMS back to CMS. Only few had changed (2.2%) or considered changing (2.2%) their AMS brand, and few (2.6%) considered replacing their AMS with parlor milking. Reasons for the latter were, e.g., that taking care of the 24/7 standby for the AMS had been too arduous for a single farmer, or it would be more flexible to gradually increase the number of dairy cows with a CMS.

## Discussion

### Automatic Milking Update

Automatic milking systems have been commercially available for almost a quarter century, and they have established a strong position in many countries (9, 10). During the past decade, the total number of AMS farms has increased notably in Finland and other Nordic countries: Sweden, Norway, Denmark, and Iceland (9).

According to annual Nordic dairy statistics, 4,293 Nordic dairy farms had AMS with 6,894 milking stalls in 2014 (9). Outside Europe, AMS has been introduced, e.g., in Canada, USA, New Zealand, and Australia (10, 16). However, there are no current sales statistics available worldwide on the number of AMS.

In the Nordic countries, AMS farms represented about 16% of all dairy farms, 28% of the dairy cows, and 29% of the total milk production in 2014 (9). Nordic AMS farms had 1.6 milking stalls and about 90 dairy cows on average (9). The Danish AMS farms were largest in the Nordic countries with the average of 2.8 milking stalls per farm (9).

Large dairy farms may acquire an AMS with several milking robots and milking stalls (one per about 60 lactating dairy cows), a mixed operation with AMS and conventional milking parlor(s) located in the same or separate dairy barns, or a hybrid milking system where a rotary milking parlor is augmented with either internal or external milking robots. AMS has become an option for a wide size spectrum of dairy farms. However, in North America, large dairy farms still rely primarily on conventional parlor milking, likely due to adequate labor supply and low labor costs.

Many AMS studies have addressed the health and welfare of dairy cows. Among others, Jacobs and Siegford (11) and Hovinen and Pyörälä (13) have reviewed these issues. Proficient knowledge and management skills of dairy farmers with an AMS have been stressed in these and other studies as well (14–16). Even though these issues were mostly out of the scope of our study, we acknowledge their importance for the progress of sustainable dairy production. However, there is only limited information on the occupational health and safety risks in AMS. There is a need for this information because the number of dairy workers involved in AMS is already substantial worldwide and growing.

### Occupational Health and Safety in Using AMS

Our anonymous online survey explored changes in working conditions when changing from conventional milking system (CMS) to AMS. This information could be used to generate recommendations for the prevention of adverse health outcomes among the present and future dairy workers in Finland and elsewhere.

Based on our results, changing to AMS reduced the perceived physical strain overall, as well as strain in various body regions. Previous studies have described physical exertion and paced, repetitive, and strenuous working motions and postures, particularly on large dairy farms with CMS (3, 5). AMS may have significant potential in the prevention of chronic musculoskeletal conditions caused by milking.

Some work tasks related to AMS caused physical strain among the participants. Handling rejected milk, daily cleaning of AMS, fetching cows to the milking stall, and training heifers and cows to use the AMS were mentioned. In addition to a specific line for rejected milk used on some AMS farms, we suggest an operator pit and a closable holding area (preferably with rubber flooring) could be of help in reducing physical strain. Soft flooring surface in the barn is beneficial for the cows as well (28).

The optimal depth and other features of an operator pit, which likely differs from that used in conventional parlor milking, should be studied further. We believe that as long as an operator pit is easy to clean, has proper stairs and a non-slippery floor, and is surrounded by safety railings, it may be of help in, e.g., daily cleaning, observation, and assisting the AMS.

Rodenburg (12) recommends free cow traffic with a closable holding area or a specific split entry holding area and rubber flooring in it. This area may be used for the daily fetched cows having problems with mobility or lameness and for training of heifers to use the AMS. In addition to Rodenburg, Lindahl et al. (4) describe methods of safe livestock handling.

Based on our results, mental stress in milking either declined or remained the same after the change to AMS. However, many farmers indicated increased mental stress from the demanding management of the AMS. The majority found that changing to AMS had reduced the monotonous, repetitive, paced, and hurried work in milking. These features of work typically cause both mental stress and physical strain, commonly reported in CMS (29, 30).

Several issues related to AMS caused mental stress. Among others, nightly AMS alarms and taking care of the 24/7 standby for the AMS were mentioned. These distinctive features of AMS are associated with each other: if a serious problem occurs with the AMS, it gives an alarm call to an assigned mobile phone. Hence, the system requires around the clock standby. In addition, many participants experienced mental stress in trusting the farm relief workers, hired labor, or both to manage with the AMS.

We suggest that in addition to workplace orientation and job guidance, vocational and continuing education of all dairy workers participating in AMS work could be of help in reducing mental stress caused by the abovementioned and other issues related to AMS. Developing and offering specific courses with emphasis on the daily management of the AMS would be advisable.

Our results regarding the perceived physical strain and mental stress are consistent with, and augment, earlier findings presented by Mathijs (21), who charted socioeconomic aspects of automatic milking among farmers (*n* = 107) in Belgium, Denmark, Germany, and The Netherlands.

We found that changing to AMS reduced the perceived injury risk in general. This reduction was most evident in the injury risk caused by working in close proximity to the hooves of the dairy cows, which is a typical risk in CMS. According to previous studies (3, 4), dairy cows’ kicks, head-butts, and tramples are some of the major causes of occupational injuries on dairy farms.

The majority of our participants mentioned AMS-related work tasks causing injury risks, such as training of heifers and dairy cows to use the AMS, assisting the milking robot, and medication or grouping of the cattle. We suggest that the previously mentioned operator pit and a closable holding area could be of help in reducing the occupational injury risk as well.

Changing to AMS reduced the risk of musculoskeletal, respiratory, and skin diseases. Common respiratory conditions among farmers include allergic rhinitis, allergic asthma, and hypersensitivity pneumonitis caused by organic dust from animals, grain, and hay (3, 31). Common skin diseases among farmers include irritant and allergic contact dermatitis caused by cow dander, moisture, dirt, rubber (e.g., in gloves and boots), disinfectants, and detergents (3, 32).

Male farmers reported reduced physical strain, mental stress, and injury risk more often than female farmers after changing to AMS. Earlier research by Karttunen and Rautiainen (7) described gender division of farm work among Finnish dairy farm couples. Results in the current study are likely affected by the gender division of specific work tasks in animal husbandry. Further studies should address the specific differences by gender in AMS and CMS work.

### Other Factors Related to Using AMS

Changing to AMS increased flexibility in the organization of all farm work, the leisure time, and the general quality of life among the majority of the participants. These positive issues may be related to each other; more freedom to shift between work and leisure time likely adds to quality of life. These findings are consistent with previous findings of Mathijs (21), Molfino et al. (22), and Bergman and Rabinowicz (33).

In addition to enhanced physical health of dairy farmers, Mathijs (21) reported improved quality of life after changing to AMS. Molfino et al. (22) conducted labor audits and surveys on Australian AMS farms (*n* = 5) and reported positive impact of the adoption of the AMS on labor and lifestyle. Among others, reduction in physical work and increased flexibility in work schedules were reported (22). Bergman and Rabinowicz (33) addressed reasons for both installing and not installing an AMS on Swedish dairy farms (*n* = 734). Among others, gaining more time for family and friends was regarded as an important reason for installing an AMS (33).

The majority of participants indicated that AMS increased the productivity of dairy work measured by produced milk liters per work hours. However, they had large variation in their number of dairy cows and annual milk production, regardless of the number of automatic milking stalls they had in use. The economic viability of AMS is compromised if the system is not fully utilized, and the productivity of work may also be low as a result. These issues should be examined thoroughly in future studies.

Most participants stated that changing to AMS enabled them as well as their hired labor and farm relief workers to have safer and healthier working conditions. They indicated that changing to AMS increased the attractiveness of dairy farming among the younger generation. Enhanced working conditions (i.e., reduced physical strain, mental stress, injury risk, and disease risk) with AMS may create more attractive workplaces for the current and future dairy workers and improve the sustainability of dairy production.

The majority of the participants had no plans of changing from AMS back to CMS. However, few dissatisfied farmers gave comments that should be paid attention to. First, being on standby around the clock for the AMS may be too tiresome for a single person if there is no substitute worker. Presumably, this issue becomes more of a problem if, e.g., the nightly alarms caused by the AMS are frequent. Second, CMS may be both technically and economically more flexible than AMS regarding the gradual increase in the number of dairy cows.

### Strengths and Limitations of the Study

The strengths of this study included covering a large variety of work-related exposures and risks and complete responses to the primary questions due to the data collection method. Reliability of this study was strengthened by including only participants with prior work experience in CMS.

The average number of dairy cows on our study farms was higher than on Finnish dairy farms with a free-stall barn in general; 82 and 55, respectively. With progressing structural change, we believe the farmers in our study population are more likely to continue their production than their peers from smaller farms.

Respondent bias (inability or unwillingness to provide accurate answers) may have affected our results. To reduce this, our survey was pre-tested and edited based on the received remarks, and an opt-out choice was included in all Likert-scale statements. Both five-point and three-point Likert scales were used, depending on the nature of the question. Anchoring descriptions at each level of the Likert scales could have improved the accuracy of the responses, but adding length to questions could have reduced the response rate.

We did not give a definition for mental stress, which can be beneficial or harmful. However, the majority of our study questions regarding mental stress charted negative effects of stress by default.

Classification of the study population based on prior work experience in CMS (pipeline, parlor, or both) could have produced more specific results. Over half of the participants had work experience in both pipeline and parlor milking. It was not possible to differentiate findings between the two types of CMS.

The low response rate (25.2%) was a limitation of our study. The high volume of record keeping and reporting burden in farming may have reduced farmers’ interest to participate in our voluntary survey. Our participants possessed 25.5% of all automatic milking stalls active in Finland at the end of 2014. Furthermore, their AMS had on average 1.4 milking stalls, identical to all Finnish AMS farms. These results indicate that our study sample was similar to all Finnish AMS farms with regards to size of the dairy herd and milking stalls per AMS.

It is possible that self-selection of the participants introduced some biases, and it is unknown which way they may have affected the results. On one hand, those with health problems, poor experiences with AMS, or both, may have greater barriers to respond to surveys. On the other hand, those satisfied with their investment in AMS may have been more interested in responding to this kind of survey.

## Conclusion

Previous studies have indicated that conventional pipeline and parlor milking expose dairy farmers and workers to various adverse health outcomes. Our study investigated the occupational health and safety risks in AMS, compared to CMS.

The results indicate that AMS may have significant potential in the prevention of physical strain and occupational injuries and diseases in milking of dairy cows. In addition, AMS may reduce certain features of work which typically cause mental stress in CMS. Enhanced working conditions and higher productivity of dairy work after changing to AMS may also improve the economic viability and sustainability of dairy production and create more attractive working places for future dairy workers.

However, certain risks in AMS require further attention with regards to occupational health and safety. These include safety in training of heifers to use the AMS, mental stress related to nightly alarms caused by the AMS, ergonomics in the handling of rejected milk, and daily cleaning of the AMS. In addition, expertise of all dairy workers using AMS requires enhancing.

We recommend the inclusion of these results to the vocational and continuing education of the current and future farmers, farm relief workers, and hired workers. In addition to formal education, repeated informing and advising is important. In doing this, positive examples from real life are advisable.

## Author Contributions

JK and RR conceived and designed the study, collected, analyzed, and interpreted the data, and drafted and wrote the manuscript. CL-K contributed to the interpretation of the data and drafting and writing the manuscript. All authors revised and approved the final manuscript.

## Conflict of Interest Statement

The authors declare that the research was conducted in the absence of any commercial or financial relationships that could be construed as a potential conflict of interest.
